# Collagenase injections for Dupuytren disease: 3-year treatment outcomes and predictors of recurrence in 89 hands

**DOI:** 10.1080/17453674.2019.1663472

**Published:** 2019-09-10

**Authors:** Jesper Nordenskjöld, Anna Lauritzson, Anna Åkesson, Isam Atroshi

**Affiliations:** a Department of Orthopedics, Hässleholm-Kristianstad Hospitals, Hässleholm;; b Department of Rehabilitation, Hässleholm Hospital, Hässleholm;; c Clinical Studies Sweden—Forum South, Skåne University Hospital, Lund;; d Department of Clinical Sciences—Orthopedics, Lund University, Lund, Sweden

## Abstract

Background and purpose — Few prospective studies have reported the long-term effect durability of collagenase injections for Dupuytren disease. We assessed the 3-year treatment outcome of collagenase injections and predictors of recurrence.

Patients and methods — We conducted a single-center prospective cohort study. Indication for collagenase injection was palpable Dupuytren’s cord and active extension deficit (AED) ≥ 20° in the metacarpophalangeal (MCP) and/or proximal interphalangeal (PIP) joint. From November 2012 through June 2013, we treated 86 consecutive patients (92 hands, 126 fingers). A hand therapist measured joint contracture before, 5 weeks, and 3 years after injection. The patients rated their treatment satisfaction. Primary outcome was proportion of treated joints with ≥ 20° AED worsening between the 5-week and 3-year measurements. We analyzed predictors of recurrence.

Results — 3-year outcomes were available for 83 of the 86 patients (89 hands, 120 treated fingers). Between the 5-week and 3-year measurements, AED worsened by ≥ 20° in 17 MCP (14%) and 28 PIP (23%) joints. At 3 years, complete correction (passive extension deficit 0–5°) was present in 73% of MCP and 35% of PIP joints. Treatment of small finger PIP joint contracture, greater pretreatment contracture severity, and previous fasciectomy on the treated finger were statistically significant predictors of recurrence. Treatment satisfaction was rated as very satisfied or satisfied in 59 of 87 hands. No long-term treatment-related adverse events were observed.

Interpretation — 3 years after collagenase injections for Dupuytren disease, improvement was maintained and treatment satisfaction reported in two-thirds of the treated hands, with no adverse events. Complete contracture correction was achieved in 3 of 4 MCP joints, but in only a third of the PIP joints.

Open surgery (limited fasciectomy) has been the most common treatment method for Dupuytren disease (Gerber et al. 2011, Dias et al. 2013, Liu et al. 2013, Nordenskjöld et al. 2017a). Collagenase injection and percutaneous needle fasciotomy are now established first-line treatment options (van Rijssen et al. 2012, Peimer et al. 2015). Advantages of these minimally invasive procedures compared with fasciectomy are safety (Krefter et al. 2017), quick recovery and lower cost (Atroshi et al. 2014).

Treatment with collagenase comprises injection into the cord, followed by finger extension 1 to 2 days later. Since the initial multicenter randomized controlled trial (Hurst et al. 2009) the procedure of injection and finger manipulation (extension) has evolved. The use of local anesthesia before the extension procedure, to optimize contracture reduction, is considered standard practice (Manning et al. 2013). Furthermore, treating multiple joints in 1 session using 2 simultaneous injections (Coleman et al. 2014, Gaston et al. 2015) and injecting a higher dose (Atroshi et al. 2015, Grandizio et al. 2017) has been shown to be safe and effective.

Few prospective studies have reported the long-term effect durability of collagenase treatment. The initial multicenter study has reported outcomes at 3 and 5 years (Peimer et al. 2013, 2015) and another study has reported outcomes at 5 years (Werlinrud et al. 2018), both studies using the original injection technique. We have previously reported treatment outcomes at 2 years using a modified injection technique (Lauritzson and Atroshi 2017). In the present study we assess 3-year treatment outcomes of collagenase injections using the modified injection technique, including assessment of predictors of recurrence and patient dissatisfaction.

## Patients and methods

### Study design and eligibility criteria

We conducted a prospective cohort study at 1 orthopedic department in Southern Sweden. This department is the only center that treats patients with Dupuytren disease in a region with 300,000 inhabitants and no patients are referred for treatment at other centers. The indication for treatment with collagenase injections was presence of a palpable cord and an active extension deficit (AED) of ≥ 20° in the metacarpo­phalangeal (MCP) joint and/or proximal interphalangeal (PIP) joint.

### Patients

From November 2012 through June 2013, we treated 86 consecutive patients (92 hands, 126 fingers) with collagenase injections. During the study period 34 patients (34 hands) were treated with limited fasciectomy in our department (see Supplementary data).

All patients were asked to participate in a follow-up examination at a minimum of 3 years after first injection. The mean age at baseline was 72 years (52–91) and 64 patients were men. 11 patients (15 fingers) had previously been treated with surgery (limited fasciectomy) on the study fingers. 7 patients with contractures in 2 or more fingers received 2 simultaneous injections (2 vials). The small finger was most commonly treated (56), followed by the ring finger (49) and middle finger (17), whereas treatment of the index finger (3) and thumb (1) was uncommon. Treatment involved the right hand in 64 of the 92 treated hands.

### Intervention

A hand surgeon injected collagenase into the cord using a modified method (Atroshi et al. 2015). After reconstituting collagenase with 0.39 mL of diluent, the surgeon injected all reconstituted collagenase (approximately 0.80 mg) in the cord, distributed in multiple spots along the palpable cord, from the PIP joint to the palmar crease. All palpable cords across the PIP joint were treated. Before the injection, local anesthesia (10 mL of 10 mg/mL mepivacaine buffered with sodium bicarbonate) was administered as a nerve block in the proximal palm (Nordenskjöld et al. 2017b). After injection, a nurse applied a soft dressing and the hand therapist gave the patient verbal and written instructions regarding edema prophylaxis (hand exercises, elevation during rest) and avoidance of heavy use of the hand.

The surgeon performed finger manipulation 1 day (43 fingers) or 2 days (83 fingers) after collagenase injection, as schedule permitted. The surgeon injected local anesthetic (similar to that before collagenase injection) and after about 20 minutes performed finger manipulation by applying pressure with the thumb along the cord to disrupt it and then manipulating the MCP and PIP joints into maximum possible extension.

Immediately after finger manipulation, the hand therapist applied a static splint with fingers in maximal possible extension and gave instructions on edema management and range of motion exercises. The patients were instructed to use the splint at night for 8 weeks. Skin tears after the manipulation procedure were treated with simple dressing and nurse follow-ups, as described previously (Atroshi et al. 2015). The patients returned to the hand therapist after 1 week for splint adjustment. In any case where contracture correction was incomplete and the patient was willing to receive further treatment, the surgeon scheduled the patient for a second injection (minimum 30-day interval).

### Measurements

Before and 5 weeks after treatment, an experienced hand therapist measured AED in the fingers with a hand-held metal goniometer and recorded the results in a standardized protocol. At 3 years after injection, a single experienced hand therapist measured both AED and passive extension deficit (PED) in the fingers. All measurements were done independently of the treating surgeon. All hands were examined for possible treatment-related complications at the 5-week and 3-year follow-up evaluations. The patients were also asked to rate their satisfaction with treatment outcome according to a 4-point scale (1: very satisfied, 2: satisfied, 3: neutral, 4: dissatisfied).

We reviewed the electronic records of all patients to ascertain any subsequent surgery or other procedures on the study hand.

### Statistics

The primary outcome was treatment effect non-durability defined as the proportion of patients that worsen by ≥ 20° in AED in a treated joint between the 5-week and the 3-year measurements. Recently an expert group on Dupuytren disease reached a consensus that defined recurrence as contracture ≥ 20° in a treated joint at 1 year compared with 6 weeks post-treatment (Kan et al. 2017). Furthermore, we considered this cut-off value to be of clinical importance since it has been used in the previous collagenase multicenter study (Peimer et al. 2015).

We recorded the mean AED values for MCP and PIP joints for all treated fingers at baseline, 5 weeks, and 3 years. Analysis of PED values was possible only for the 3-year follow-up, because only AED was measured at baseline and at 5 weeks. We also calculated the proportion of joints with a PED value 0–5°, defined as complete correction by previous studies, including only treated joints with baseline AED ≥ 10° (to avoid overestimating the correction). Hyperextension was considered as 0° extension deficit. The changes in AED between baseline, 5 weeks, and 3 years were statistically tested with the paired t-test. Predictors of recurrent contracture (worsening by ≥ 20° in AED) at 3 years were analyzed with a mixed-effects logistic regression model adjusting for sex and age, and odds ratios (OR) were calculated separately for MCP and PIP joints. In this analysis we excluded thumb and index finger joints due to small number of treated fingers (n = 3). Predictors of patient dissatisfaction at 3 years were analyzed with a Cox regression model adjusting for baseline factors and relative risks (RR) were calculated for total AED (MCP plus PIP). For patients who underwent limited fasciectomy after collagenase injection (n = 2) the preoperative contracture values were used in the analyses. We present the data as proportions and means with standard deviations (SD) or 95% confidence intervals (CI). A 2-sided p-value < 0.05 indicate statistical significance. The analyses were performed with Stata (version 14; StataCorp, College Station TX, USA) and SPSS (version 24; IBM Corp, Armonk, NY, USA).

### Ethics, funding, and potential conflicts of interest

This research was reviewed by the Regional Ethical Review Board in Lund (2013/656) and was conducted in accordance with the Helsinki Declaration of 1975 as revised in 2000. All patients received verbal and written information about the study and gave informed consent. The research was supported by Region Skåne. Author IA was a member of an expert group on Dupuytren disease for Pfizer in 2012 and participated in meetings organized by Sobi. The manufacturer did not support the study or any of the authors.

## Results

At the 3-year follow-up, 3 patients (2 men) were deceased. Thus, 3-year outcomes were available for 83 of the patients (89 hands, 120 treated fingers).

### Joint contracture

Between the 5-week and 3-year measurements, AED worsened by ≥ 20° in 17 MCP, 28 PIP, and either joint in 41 fingers. For all treated fingers, the mean total AED (SD) was 75° (39) before injection, 21° (26) at 5 weeks, and 32° (29) at 3 years. The corresponding values for MCP joints were 44° (25), 9° (15), and 12° (17), and for PIP joints 31° (29), 12° (17) and 20° (24), respectively (Table 1). At the 3-year follow-up complete correction (PED 0–5°) in treated joints with baseline AED ≥ 10° was observed in 79 of 108 MCP and 25 of 72 of PIP joints.

**Table 1.** Active extension deficit before (baseline), 5 weeks, and 3 years after injection. Values are mean degrees (SD) unless specified otherwise. Number of fingers (n) in 86 patients (83 patients at 3 years)

				Mean difference (CI)	
	Baseline n = 126	5 weeks n = 126	3 years n = 120	Baseline–5 weeks	5 weeks–3 years
MCP	44 (25)	9 (15)	12 (17)	35 (31–39) **^a^**	–4 (–6 to –1) **^b^**
PIP	31 (29)	12 (17)	20 (24)	19 (15–22) **^a^**	–9 (–12 to –6) **^a^**
Total	75 (39)	21 (26)	32 (29)	54 (49–58) **^a^**	–13 (–16 to –9) **^a^**

CI: 95% confidence interval, MCP: metacarpophalangeal,

PIP: proximal interphalangeal.

**^a^**p < 0.001, **^b^**p = 0.002.

Treatment of small finger PIP joint contracture, greater severity of pretreatment contracture and previous fasciectomy on the treated finger were statistically significant predictors of contracture recurrence (Table 2). The OR (CI) for ring finger PIP joint contracture (vs. small finger) was 0.20 (0.05–0.72), for increasing baseline severity (per degree) of MCP contracture 1.06 (1.03–1.09) and of PIP contracture 1.08 (1.05–1.12), and for previous fasciectomy (MCP joint) 7.2 (1.4–39). Age and sex had no significant association with recurrence.

**Table 2.** Predictors of recurrence at 3 years. Mixed effects regression model (data from 117 treated fingers, excluding thumb and index finger)

Variable (referent category/unit)	Odds ratio (95% confidence interval)	
	MCP	PIP
Sex (male)	2.2 (0.5–9)	4.1 (1.1–15)
Age (per year)	1.03 (0.95–1.11)	1.08 (1.01–1.16)
Finger (small)		
Ring	3.1 (1.1–9)	0.2 (0.05–0.7) **^a^**
Middle	5.8 (1.3–27)	0.3 (0.05–1.9)
Baseline contracture (per °)	1.06 (1.03–1.09) **^b^**	1.08 (1.05–1.12) **^b^**
Recurrence after fasciectomy (no)	7.2 (1.4–39) **^c^**	3.2 (0.7–16)
Injection-extension interval (1 day)	0.9 (0.3–3)	1.0 (0.3–2.9)

MCP: metacarpophalangeal, PIP: proximal interphalangeal.

**^a^**p = 0.01, **^b^**p < 0.001, **^c^**p = 0.02.

### Patient satisfaction

Patients rated degree of satisfaction with outcome in 87 of the 92 treated hands: very satisfied in 39 hands, satisfied in 20 hands, neutral in 12 hands, and dissatisfied in 16 hands. Mean improvement in total AED (SD) from baseline to 3 years was 47° (34) among very satisfied and satisfied patients and 22° (22) among dissatisfied patients; mean difference 25° (CI 13–37). Patient dissatisfaction was higher with increasing pretreatment contracture severity (RR 1.02, CI 1.01–1.04) and with contracture recurrence (RR 1.03, CI 1.02–1.05). Age and sex had no significant association with dissatisfaction.

### Reinjections, subsequent surgery, and adverse events

4 patients received a second injection during the study period (at 4, 8, 12, and 24 months after the first injection, respectively). 2 other patients underwent limited fasciectomy (at 6 months and 3 years after injection, respectively). No other patients had any other surgical interventions during the study period. The occurrence of skin tears was recorded and published in a previous study (Atroshi et al. 2015). At the 5-week and the 3-year follow-up the hand therapist did not observe, and the patients did not report, any adverse events. No patient suffered from neurovascular injury, flexor tendon injury, infection, or complex regional pain syndrome during the study period.

## Discussion

This prospective cohort study of consecutive patients with Dupuytren disease treated with collagenase injection using a modified injection method, with near complete follow-up, has shown that improvement was maintained in two-thirds of the treated hands 3 years after treatment. Complete contracture correction, defined as PED 0–5°, was achieved in 3 of 4 MCP joints but in only a third of the PIP joints. Previous studies have also shown that PIP joints have a higher recurrence rate regardless of treatment method (van Rijssen et al. 2012, Peimer et al. 2015, Hansen et al. 2017). Furthermore, the analyses identified PIP joint contracture in the small finger, higher severity of pretreatment contracture, and previous surgical fasciectomy as predictors of worsening after initial correction. Although the majority of patients (about two-thirds) were satisfied with the treatment, 18% were dissatisfied. These results are similar to a previous study examining satisfaction after collagenase treatment (Bradley and Warwick 2016), and were correlated with treatment outcome and severity of pretreatment contracture. Identifying predictors of recurrence and patient dissatisfaction would be helpful for surgeons when informing patients with regard to treatment expectations. At 3 years, no adverse events were noted by the evaluating hand therapist or reported by the patients, indicating that collagenase injection is a safe treatment method in the long term.

In the Collagenase Option for Reduction of Dupuytren Long-Term Evaluation of Safety Study (CORDLESS), 643 of 950 of the initial study participants could be evaluated 3 years after treatment (Peimer et al. 2013). At 3 years, “worsening” of contracture (defined as ≥ 20° increase in contracture in fully or partially corrected joints with or without a palpable cord, or subsequent treatment) was 28% for MCP joints and 58% for PIP joints. In our study, using the same definition, recurrence occurred in 14% of MCP joints and 23% of PIP joints. A study of 47 patients with isolated MCP joint contracture (McFarlane et al. 2016), treated with a single 0.58 mg collagenase injection, reported a recurrence (defined as contracture > 20°) in 12 patients at 2 years. A study of 68 patients with both MCP and PIP joint contractures evaluated 2 years after collagenase injection (Van Beeck et al. 2017) showed ≥ 20° increase in extension deficit in 11 of 39 MCP joints and 18 of 29 PIP joints. Treatment outcome in our study is better, which may have several explanations. First, the use of the modified method injecting multiple spots in the cord and use of a higher dose. Second, in comparison with the CORDLESS study, use of local anesthesia before finger extension procedure may enhance optimal contracture reduction. Furthermore, we have shown a high prevalence of skin tears after finger manipulation (40% of treated hands) in a previous study (Atroshi et al. 2015), but all wounds healed without complications. Therefore, our aim during the finger manipulation procedure is always to achieve the best possible contracture reduction despite skin tear occurrence. In our previous prospective study reporting the 2-year outcome of collagenase injections in 48 patients (Lauritzson and Atroshi 2017), recurrence (AED worsening ≥ 20°) was observed in 7 of 50 MCP joints, similar to the 3-year outcome, but was lower for the PIP joint (7 of 50 PIP joints).

There are few randomized controlled trials comparing treatment methods for Dupuytren disease. A recent Cochrane review states that there is insufficient evidence to show the relative superiority of different surgical procedures (Rodrigues et al. 2015). In a randomized controlled trial comparing percutaneous needle fasciotomy and limited fasciectomy (van Rijssen et al. 2012), with recurrence defined as ≥ 30° in total PED from 6 weeks to 3 years, the authors reported recurrence in 4 of 46 treated hands in the limited fasciectomy group and 35 of 55 hands in the needle fasciotomy group. Using the same definition, recurrence in our study occurred in 19 of 89 hands.

Randomized controlled trials comparing collagenase injection with percutaneous needle fasciotomy for isolated MCP joint contractures have been reported with up to 3-year follow-up. These studies show no statistically significant difference in treatment outcome, and the authors suggest an advantage for percutaneous needle fasciotomy, foremost based on presumed difference in initial treatment cost (Scherman et al. 2018, Strömberg et al. 2018). Our data show that during the study period 34 hands were treated with surgical fasciectomy at the study center, constituting 27% of all treatments for Dupuytren disease (see Supplementary data). A similar treatment trend of decreasing use of fasciectomy was observed in the United States after the introduction of collagenase treatment (Zhao et al. 2016). The finding may suggest that collagenase injections can replace limited fasciectomy to a large extent. Since surgical fasciectomy is associated with higher costs than treatment with collagenase (Atroshi et al. 2014, Sefton et al. 2018), the number of fasciectomy procedures performed has to be considered in an overall cost-effectiveness analysis of treating Dupuytren disease.

In a randomized study comparing percutaneous needle fasciotomy and collagenase injection for isolated PIP joint contractures (Skov et al. 2017), treatment efficacy was similar at 2 years. However, in that study 28 of 29 of the patients treated with collagenase had a PIP joint contracture in the small finger, compared with only 15 of 21 patients in the needle fasciotomy group. In our study we have identified small-finger PIP joint contracture as a predictor of recurrence. This finding suggests that future randomized controlled trials should be stratified according to small finger involvement.

The limitations of our study include a single center and moderate sample size, implying uncertain generalizability. In comparison with other studies using PED as the primary outcome measure, we measured AED before treatment and 5 weeks after treatment since it may be less examiner dependent (Nordenskjöld et al. 2018) and may be more relevant as a measure of hand function. Thus, our posttreatment AED values are conservative compared with posttreatment PED values in other studies. We added the measurement of PED at the 3-year evaluation to enable comparison with other studies. Another limitation is the lack of follow-up between 5 weeks and 3 years. Furthermore, patient-related outcome measures (PROMs) are limited to a satisfaction scale in this study, and more information regarding the patients’ view on collagenase treatment could have been obtained with the addition of more PROMs. However, the disabilities of the arm, shoulder, and hand (DASH) questionnaire has shown only modest responsiveness in Dupuytren disease (Rodrigues et al. 2016), and disease-specific measures, such as Unité Rhumatologique des Affections de la Main (URAM) scale (Beaudreuil et al. 2011) need further independent validation.

The major strength of our study is the high participation rate at the 3-year evaluation, with data available for 100% of the treated hands of patients still living. The study center, an orthopedic department to which the vast majority of patients seeking care for Dupuytren disease are referred, enhances generalizability. Furthermore, all baseline and follow-up measurements of treatment outcomes were performed using a standardized protocol and independently of the treating surgeon.

In summary, our prospective cohort study with near complete follow-up is 1 of the largest studies that reports collagenase treatment outcomes at 3 years. Complete contracture correction was maintained in 3 of 4 MCP joints, but only in a third of PIP joints. It shows that collagenase injection using a modified injection technique is a safe treatment method for Dupuytren disease, with treatment satisfaction reported in two-thirds of the treated hands.  

## Supplementary Material

Supplemental Material

## Supplementary data

Supplementary data are available in the online version of this article, http://dx.doi.org/10.1080/17453674.2019.1663472
JN: Data acquisition and analysis, drafting of the manuscript. AL: Data acquisition. IA: Study design and conception, data acquisition and analysis, revision of the manuscript. *Acta* thanks Michel E H Boeckstyns, Joakim Strömberg, and David Warwick for help with peer review of this study.
