# Supplementary material for: Collagenase injections for Dupuytren disease: 3-year treatment outcomes and predictors of recurrence in 89 hands
Source: Acta Orthop. 2019 Sep 10;90(6):517–22. doi: 10.1080/17453674.2019.1663472 (PMC6844429; doi:10.1080/17453674.2019.1663472)
Supplement: Supplemental Material [file IORT_A_1663472_SM8389.pdf]

Supplementary data

Patients treated with surgery (limited fasciectomy) at the study center during the study period (November 2012 through June 2013)

Treated patients	34 (34 hands)
Mean age (range), years	68 (49–84)
Men, n	28 (82%)

Reasons for choosing surgery (as stated in patient's electronic records)

Recurrence after previous	
Limited fasciectomy	6
Percutaneous needle fasciotomy	4
Collagenase injection	1
Failed percutaneous needle fasciotomy	2
Severe proximal interphalangeal joint contracture	5
Patient's choice	5
Surgeon's preference (only performs surgery)	11
